# Flexible and Versatile as a Chameleon—Sophisticated Functions of microRNA-199a

**DOI:** 10.3390/ijms13078449

**Published:** 2012-07-09

**Authors:** Shen Gu, Wai-Yee Chan

**Affiliations:** School of Biomedical Sciences, Faculty of Medicine, The Chinese University of Hong Kong, Shatin, N.T., Hong Kong, China; E-Mail: gushen0419@gmail.com

**Keywords:** microRNA, targets, miRNA-199a

## Abstract

Although widely studied in the past decade, our knowledge of the functional role of microRNAs (miRNAs) remains limited. Among the many miRNAs identified in humans, we focus on miR-199a due to its varied and important functions in diverse models and systems. Its expression is finely regulated by promoter methylation and direct binding of transcription factors such as TWIST1. During tumorigenesis, depending on the nature of the cancer, miR-199a, especially its -3p mature form, may act as either a potential tumor suppressor or an oncogene. Its 5p mature form has been shown to protect cardiomyocytes from hypoxic damage via its action on HIF1α. It also has a functional role in stem cell differentiation, embryo development, hepatitis, liver fibrosis, *etc*. Though it has varied biological activities, its regulation has not been reviewed. The varied and protean functions of miR-199a suggest that efforts to generalize the action of a miRNA are problematic. This review provides a comprehensive survey of the literature on miR-199a as an example of the complexity of miRNA biology and suggests future directions for miRNA research.

## 1. Introduction

Until the present time, the identification of certain small RNA molecules as microRNA (miRNA) has been based on its structure/size. Criteria include hairpin precursor structures, phylogenetic conservation in multiple species, and experimental documentation of a small RNA molecule [1]. The most recent miRBase released in November 2011 identified 21,643 mature miRNA products in 168 species. More than 2,000 were shown to exist in humans [2]. It is becoming clear that not all miRNAs are functionally important [3]. The well-conserved miR-199a, identified by diverse high-throughput screenings in many systems, suggests it may have important and comprehensive functions in different models.

## 2. miR-199a: One among Thousands

In 2003, two mature forms derived from the same precursor, miR-199-s (from the 5′ half) and miR-199-as (from the 3′ half), were cloned from human osteoblast sarcoma cells and mouse skin, respectively [1]. That same year, the identity of miR-199a was computationally predicted, based on its conservation among human, mouse and puffer fish [4]. Expression of the microRNA was validated in zebrafish, and its ends mapped by cloning. The two microRNA sequences were named miR-199a and miR-199a* (from the 3′ arm), respectively. Later it was shown that both mature forms are expressed in humans, and it was renamed miR-199a-5p and miR-199a-3p, respectively [5]. There are two loci that encode the precursor of miR-199a-5p and -3p in the human genome; one is on Chromosome 1 (miR-199a-2, miRBase Accession MI0000281) and the other on Chromosome 19 (miR-199a-1, miRBase Accession MI0000242).

### 2.1. Regulation of miR-199a Expression

As shown in UCSC genome browser, miR-199a-1 located on Chromosome 19 (Chr19) is embedded in the anti-sense strand of intron 15 of Dynamin 2 (*DNM2*), whereas miR-199a-2 located on Chromosome 1 (Chr1) is embedded in the anti-sense strand of intron 14 of Dynamin 3 (*DNM3*). There is no evidence of functional correlation between the expression of the dynamin genes and the miR-199a precursors: this may be due to the fact that the expression of the miRNA precursors is controlled by their own promoters.

The *DNM3* locus encodes an expressed anti-sense transcript, *DNM3OS*, which stands for *DNM3* opposite strand. *DNM3OS* gives rise to miR-199a-2 and another microRNA, miR-214. Sometimes the two miRNAs are referred to as members of the miR199a-2/214 cluster [6]. Comparison of *DNM3OS* with its mouse homolog *Dnm3os* [7] indicated the human and mouse miR-199a-2 and miR-214 are highly homologous (99% and 100%, respectively). In mouse, *Dnm3os* was shown to be a miRNA-encoding gene that is indispensable for normal skeletal development and body growth [8]. The high sequence homology between human and mouse suggests *DNM3OS*, in the form of miR-199a-2 and miR-214, may play important roles in human growth and development.

**Figure 1:**
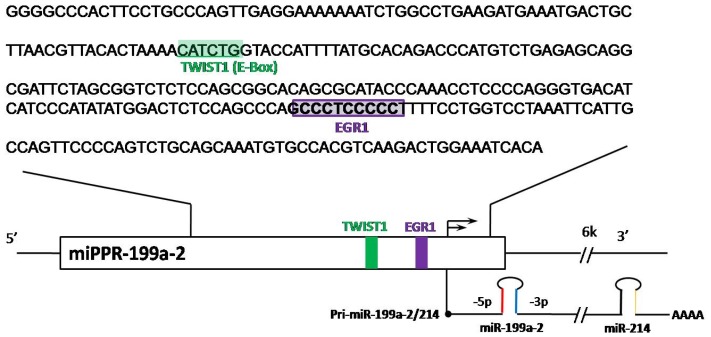
Promoter sequence of miR-199a-2 on Chr1 with binding sites for TWIST1 and EGR1 highlighted. (Adapted from References [6] and [13]).

Currently, two mechanisms that control the expression of miR-199a have been discovered. One is the regulation by transcription factors TWIST1 and EGR1 on Chr1; the other is the methylation status of miR-199a promoters on both Chr1 and Chr19. Since there is no clearly defined “promoter region” for miR-199a-1, the region containing several hundred base pairs upstream of miR-199a-1 was considered as its promoter region. For miR-199a-1 on Chr19 there is a predicted CpG island between ~130 and 540 bp upstream of the mature miR-199a sequence [9]. For miR-199a-2 on Chr1 the promoter region is relatively CpG poor, and a putative promoter region (miPPR-199a-2) of 1349 bp starting 81 bp upstream of the 5’end of the miRNA hairpin region has been identified [10] (Figure 1). Studies in several cell lines showed that both promoter regions on Chr1 and Chr19 were hypermethylated (higher than 90%) in cancer cells but hypo- or not methylated in normal fibroblasts. Correspondingly the expression of miR-199a was higher in normal fibroblasts than cancer cells [9]. Genome-wide DNA methylation profiling revealed that the promoter region of miR-199a-2 on Chr1 was hypermethylated in testicular germ cell tumors compared to the hypomethylation status in normal testicular fibroblasts. Apparently hypermethylation in testicular cancer cells caused severely reduced expression of miR-199a [11]. Similar observations were made in non-small cell lung cancer, colorectal cancer and breast cancer cell lines [12].

Mouse studies showed that the expression of *Dnm3os* was regulated by the transcription factor TWIST [14]. The effect of TWIST on *Dnm3os* was tissue- and development-stage-specific albeit TWIST did not seem to be the sole factor regulating the expression of *Dnm3os*. It was later shown in human cells that TWIST1 bound to an E-box region drives the expression of the DNM3OS transcript (Figure 1), which gives rise to miR-199a-2 and miR-214 [6]. It is reasonable to speculate, similar to what has been observed in the mouse, that *DNM3OS* is directly controlled by TWIST1 under specific circumstances. As a result TWIST1 also determines the expression of miR-199a-2. Co-expression of miR-199a-2 and miR-214 has been observed in various systems, e.g., in zebrafish embryonic development [15], morphogenesis in skin (both highly expressed in hair follicle) [16], in response to stress and cardiac hypertrophy (both up-regulated) [17], in primary CNS lymphomas (both down-regulated) [18] and in antiviral responses [19].

Another transcription factor, EGR1 (Figure 1), was demonstrated to occupy the miR-199a-2/miR-214 gene promoter (one specific region in miPPR-199a-2) and induce its expression in certain cancer cells [13]. Interestingly, a direct target of both miR-199a-5p and -3p, BRM, was found in the various cancer cells. BRM in turn had a negative regulatory effect on EGR1. As a result, miR-199a and BRM formed a double negative feedback loop through EGR1. There were two distinct types of cancer cells, one with high expression of BRM (e.g., non-small cell lung cancer A549 and NCI-H1299, breast ductal cancer MDA-MB435, cervical cancer HelaS3, and oral cancer KB) while the other with very low levels of BRM (e.g., adrenocortical cancer SW13, gastric cancer AZ521, non-small cell lung cancer NCI-H522, cervical cancer C33A, and embryonic cancer cells PA-1). This phenomenon may offer possible explanations for the variable (high or low) expression of miR-199a-5p and -3p in different cancers.

Besides TWIST1, EGR1, and DNA methylation, other factors have been reported to control expression of miR-199a. Reduced expression of miR-199a-3p in hepatocellular carcinoma was shown to be mediated by histone modification and was independent of DNA methylation [20]. *In vitro* cell model studies of liver injury and fibrosis showed that farnesoid X receptor (FXR) could negatively regulate miR-199a-3p at the post-transcriptional level [21]. In mice cardiac myocytes, miR-199a-5p was upregulated during cardiac hypertrophy via β-adrenergic receptor (β-AR) stimulation, but downregulated by AKT activation during hypoxia [22]. Again, the down-regulation by AKT of miR-199a-5p was shown to be post-transcriptional [23]. Another transcription factor, signal transducer and activation of transcription 3 (STAT3) was demonstrated to negatively regulate miR-199a-2 by suppressing its promoter activity in mice cardiocytes [24]. Transfection of bone morphogenic protein 2 (BMP2) into mice mesenchymal fibroblast-like cells showed that expression of miR-199a-3p was significantly inhibited at 5 h (which was the early stage of chondrogenesis), and increased at 24 h and remained high [25].

As demonstrated by the studies in different models, the expression patterns of miR-199a in different systems are complicated and delicately regulated. These phenomena indicate that the multi-functional and versatile characteristics of miR-199a are, at least partly, fine-tuned by its source and origin.

### 2.2. miR-199a in Tumorigenesis

Extensive research has been done on miR-199a in cancers revealing its diverse expression patterns and functions in different cancer types (Table 1). It could be down-regulated as a potential tumor suppressor in some cases, or could be up-regulated as an oncogene in others. Such dramatic differences might be due to its complicated expression control mechanisms as discussed above, and its involvement in different cellular behaviors might be due to the diverse nature of its downstream targets.

#### 2.2.1. Down-Regulation of miR-199a

The most widely used cancer model in studying miR-199a is liver cancer. MiR-199a was frequently downregulated in human hepatocellular carcinoma (HCC) [31]. HIF-1α was identified as a direct target. MiR-199a inhibited cell proliferation in both *in vitro* and *in vivo* assays [32]. Another study in HCC showed that the decrement of miR-199a-3p significantly correlated with poor survival of patients, and it could target tumor-promoting PAK4 to suppress HCC growth through inhibiting the PAK4/RAF/MEK/ERK pathway both *in vitro* and *in vivo* [20]. In a study of seven HCC cell lines, in spite of the fact that all cells showed down-regulation of miR-199a-3p only two CD44+ cell lines were sensitive to the anti-proliferation and anti-invasion effects of knockin in expression of pre-miR-199a-3p. CD44+ was also shown to be a direct target of miR-199a-3p in HCC cells [33]. A similar observation on HCC studies showed that the anti-invasion effect of miR-199a-5p on its direct target DDR1 varied among individuals and cell lines [34]. More than 50% of HCC tissues and cells showed significant down-regulation of miR-199a-5p, with increased expression of the pro-invasion molecule DDR1. In addition, miR-199a-3p was shown to be a modulator of cell cycle. It sensitized the cells towards drug treatment through its target mTOR [35].

Aside from the down regulation in HCC, miR-199a also showed reduced expression in other cancers. In serous ovarian cancer patient tissues, miR-199a was down-regulated [43] and significantly correlated with a poor prognosis [44] and tumor progression [45]. However, in microcystins (MCs)-induced mice with ovarian cancer, miR-199a-3p showed increased expression [53]. The reason for this difference is unclear. In renal cell cancer (RCC), a decreased expression of miR-199a in eight RCC cell lines and 59% tissues samples (32 of 54) was found. This down-regulation of miR-199a in RCCs was correlated with higher tumor stage and nuclear overexpression of GSK-3β, which was confirmed to be a target of miR-199a [26]. In osteosarcoma cell lines and tissues, miR-199a-3p showed reduced expression. In addition, it showed tumor suppressive abilities *in vitro* by affecting proliferation, migration and cell cycle progression. Molecules that were affected and which might be targets of miR-199a-3p include MET, mTOR and STAT3 [38]. In testicular germ cell tumors, miR-199a (both -3p and -5p) showed reduced expression. Over-expression of the miRNA suppressed cancer migration, invasion and anti-proliferation. PODXL was identified as a direct target of miR-199a-5p, knockdown which suppressed cancer invasion [40]. In breast cancer, miR-199a was significantly under-expressed, and the relative expression was correlated with tumor grade and sex hormone receptor expression [41]. In bladder cancer, miR-199a-3p was down-regulated, and worked as a tumor suppressor by targeting KRT7 [49].

#### 2.2.2. Up-Regulation of miR-199a

Contrary to the down-regulation of miR-199a in HCC in adults, this miRNA showed up-regulation in pediatric hepatoblastoma patients [37]. In mature ovarian cancer stem cells, high expression of miR-199a-2 due to stimulation by TWIST1 down regulated IKKβ, therefore shutting down the IKKβ/NFκB pathway [7]. MiR-199a was expressed at higher levels in gastric cancer tissues than in normal gastric tissues; and higher in metastatic than non-metastatic gastric tissues. The miRNA positively regulated gastric cancer cell proliferation, migration and invasion. One direct target in this system was MAP3K11 [27]. Another study on gastric cancer showed that patients who remained free of recurrence for at least three years after surgery had significantly lower levels of miR-199a-3p than patients who had a recurrence [28]. Studies on Japanese gastric patient samples showed that up-regulation of miR-199a was useful as a progression-related signature [29].

Comparison between melanoma of young and older adults (>60 years old) showed increased expression of miR-199a which was speculated to regulate the TLR-MyD88-NFκB pathway [47]. In uveal (ocular) melanoma patients, both miR-199a-3p and -5p significantly discriminated the high metastatic group from the low metastatic group, with higher expression in the former [48].

There is significantly higher expression of miR-199a-3p in patients with malignant biliary tract cancer than patients with benign tumor [30]. Up-regulation of both miR-199a-3p and -5p predicted a worse prognosis in esophageal adenocarcinoma patients [39]. In Sezary Syndrome (SzS) patients (T-cell lymphoma), expression of miR-199a-3p was up-regulated, which in turn inhibited the expression of *EVL. EVL* is the host gene of miR-342 which was down-regulated in SzS patients [42], and miR-342 is known to induce apoptosis. Comparing specimens from invasive squamous cell carcinomas to normal cervical squamous epithelial tissues showed over-expression of miR-199a. Silencing of miR-199a *in vitro* using siRNA reduced cell growth indicated the pro-proliferation characteristics of miR-199a in cervical cancers [51]. Comparing two sets of acute myeloid leukemia (AML) patients, miR-199a was expressed much higher in patients with worse overall and event-free survival. High expression of miR-199a was also identified in AML patients with isolated trisomy 8 [52].

#### 2.2.3. Other Features of miR-199a in Cancer

One important observation in studying miR-199a in tumors is that subtypes of one cancer could exhibit different expression patterns of miR-199a. For example, comparison between two forms of primary CNS lymphomas, diffuse large B-cell lymphomas (DLBCL) is associated with lower expression whereas nodal DLBCL had higher expression of miR-199a [18]. Expression of miR-199a-3p was also different among different subtypes of breast cancer [54]. Expression of miR-199a in bronchial squamous carcinomas was found to be stage specific, meaning miR-199a was up-regulated in squamous cell carcinoma as compared to severe dysplasia and *in situ* carcinoma [50].

Some other *in vitro* studies of knock-in or knock-out of miR-199a in cells revealed more details on its function. MiR-199a-3p promoted proliferation and survival of endothelial cells and breast cancer cells by inhibiting its direct target Caveolin-2 [55]. In several non-small cell lung cancer (NSCLC), breast cancer (BRC) and colorectal cancer cell lines, miR-199a had a pivotal role in tumorigenesis affecting activities such as tumor growth, migration, invasion and *in vivo* distant metastasis by directly targeting AXL [12]. MiR-199a-3p has been shown to be one of the miRNAs that targeted MET at mRNA level in several cancer cell lines. Reduced expression of miR-199a-3p led to higher expression of MET and enhanced invasion [56]. Besides MET, its downstream effector ERK2 was also shown to be inhibited by miR-199a-3p, indicating the anti-proliferation, motility and invasive capabilities of the miRNA in these tumor cells [9].

Drug treatment of cancer cells exhibited expression changes of miR-199a. Phorbal 12-myristate 13-acetate (TPA)-induced differentiation of leukemia HL-60 cells, down-regulated miR-199a-3p [57]. The metabolite of oltipraz, a cancer chemopreventive drug, inhibited HIF-1α due to increased expression of pre-miR-199a-5p in colon cancer cells [58].

Besides research on human patient samples and cells, the biological effects of miR-199a in tumorigenesis have been studied in animal models. Induction of oral carcinoma by 7,12-dimethyl-benz[a]anthrance treatment in the Syrian hamster resulted in up-regulation of miR-199a [59]. Deep sequencing of miRNAs in chicken embryo fibroblasts infected with Marek’s disease virus showed lowered expression in formed tumors [60]. Conditioned knockout of *Pten* led to development of endometrial cancer with reduced expression of miR-199a-3p which targeted COX-2 [61].

MiR-199a plays important and crucial rules in tumorigenesis of a variety of systems. Whether it facilitates or inhibits formation is quite complicated and must be analyzed in the individual circumstance. In addition, miR-199a-3p has multiple targets in different cancer types (especially with cancer cells that are CD44 positive) and appears to play a more dominant role than miR-199a-5p [7].

### 2.3. miR-199a in Hepatitis, Liver Fibrosis and Its Antiviral Effects

In liver samples from patients with hepatitis C virus (HCV) infection, and mouse fibrosis livers induced by CCL4, both miR-199a-3p and -5p were up-regulated in a fibrosis progression-dependent manner [62]. Detailed research showed that activated FXR protected liver cells from injury through the induction of LKB1. However, in fibrotic livers, lowered expression of FXR resulted in elevated miR-199a-3p, which in turn suppressed the expression of its direct target LKB1 [21].

Although up-regulated in HCV infected livers, *in vitro* studies showed that miR-199a-3p could inhibit HCV genome replication, suggesting its potential antiviral role [63]. The viral replication effects of miR-199a-3p were also demonstrated for hepatitis B virus (HBV) [64]. Besides interaction with viral elements, its antiviral effects were shown to be caused by the down-regulation of several pathways including ERK/MAPK signaling, prostaglandin synthesis, oxidative stress signaling and PI3K/AKT signaling [19]. Studies on steatohepatitis indicated that mice fed with different diets to induce alcoholic or non-alcoholic fatty livers showed different miR-199a-3p profiles, with down-regulation in the former and up-regulation in the latter [65].

### 2.4. miR-199a in Cardiogenesis

The functions of miR-199a, mostly its -5p mature form in cardiomyocytes has been relatively well studied. Research on mice with cardiomyocyte-restricted knockout of STAT3 identified the pathophysiological relationship between reduced STAT3 protein levels, increased miR-199a-5p expression, and decreased expression of two direct targets, ubiquitin-conjugating enzymes UBE2G1 and UBE2I. Impairment of these enzymes results in disrupted cardiomyocyte sarcomere structure and function [24]. Studies in mouse heart cells showed that miR-199a-5p was sensitive to low oxygen levels and rapidly reduced to undetectable levels, thereby releasing its targets from its inhibitory effect. Two molecules, HIF1α and SIRT1, were identified as its direct targets. They play important controlling and regulatory roles during hypoxia or hypoxia preconditioning [66]. The rapid down-regulation of miR-199a-5p during hypoxia preconditioning was controlled by the activation of AKT pathway, which could be counteracted by activation of β-adrenergic signaling [22]. Northern blot analysis in different rat tissues showed that miR-199a was mainly expressed in lung and heart, with dominant expression in cardiomyocytes. Its expression was up-regulated in hypertrophic rat hearts. MiR-199a was essential for the maintenance of cardiomyocytes cell size. HIF1α was identified as a direct target [67]. Similar observations were made in human heart hypertrophy and failure studies, and overexpression of miR-199a resulted in elongated myocytes [17].

A detailed study of cell-specific expression of miR-199a in mouse heart, utilizing *in situ* hybridization and immunohistochemistry, demonstrated increased expression through embryogenesis. MiR-199a was localized in connective tissue cells of the heart instead of cardiomyocytes as previously reported [68]. This difference might be explained by the different focus of the studies. For example, in the *Stat3-*KO study whole mouse heart but not isolated cardiomyocytes was studied using miRNA microarrays. Regardless of these differences, miR-199a played important roles regulating heart functions. The master regulator in hypoxic conditions, HIF1α, requires a rapid increase in protein production, usually within minutes of exposure to hypoxia. The promptness of the response is vital to reduce cell damage; miR-199a-5p provides a flexible and fast means for controlling the protein level of HIF1α without changing its transcription [23]. Besides the heart, the reduced expression of miR-199a-5p during hypoxia was also observed in human pulmonary and brain epithelial cells, with increased expression of its targets FLAP at both mRNA and protein levels [69].

### 2.5. miR-199a in Osteogenesis, Chondrogenesis and Adipogenesis

Following introduction of BMP-2 into murine mesenchymal stem cells (MSCs) to stimulate its differentiation, miR-199a-3p was up-regulated and acted as a negative regulator of early chondrogenesis via its direct target SMAD1, a transcription factor that enhances osteogenesis [25]. Another study of mouse bone marrow stromal cells chondrogenesis gave similar results, with more than 10-fold up-regulation of miR-199a. The study also predicted HIF1α to be its direct target and an important regulator involved in the process [70]. Studies in human MSCs showed induction of miR-199a expression during both osteogenic and adipogenic differentiation, and together with other miRNAs, decreased the expression of LIF [71].

Studies in human osteoarthritis (OA) chondrocytes indicated that miR-199a-3p directly targeted COX-2 mRNA. Upon IL-1β stimulation, expression of miR-199a-3p and COX-2 was inversely affected, indicating that miR-199a-3p might be an important regulator of human cartilage homeostasis and suggested potential therapeutic strategies for the treatment of OA [72]. Analysis of different sources of human MSCs indicated that miR-199a was expressed at a lower level in abdominal adipose tissue MSCs as compared to facially-derived MSCs [73]. The level of expression was also lower in aged adipose tissue derived MSCs [74].

### 2.6. miR-199a in Embryonic Stem Cells Differentiation and Embryo Development

Expression of miR-199a exhibited differences before and after stem cell differentiation. For example, after differentiation of human embryonic stem cells (hESCs) into pancreatic islet-like cells, miR-199a showed up-regulation [75]. Studies in ovarian cancer stem cells showed that the two subtypes of epithelial ovarian cancer (EOC) stem cells, type I/CD44+ and type II/CD44-, have a very distinct expression pattern of miR-199a, with type II levels being much higher. Type II EOC stem cells were the differentiated mature population of the type I EOC stem cells [7]. How miR-199a was involved is unclear.

In addition to stem cell differentiation, miR-199a also exhibited functions in embryo development. MiR-199a was shown to be indispensable in normal skeletal development and body growth in mammals [8]. Disruption of the expression of *Dnm3os* which induces miR-199a and miR-214 expression in mice resulted in skeletal defects. Besides embryo development, studies of the implantation process in mice showed that miR-199a-3p exhibits spatiotemporally coincident expression with *Cox-2* in the uterus. *Cox-2* is a gene critical for implantation and is directly regulated by miR-199a-3p [76]. MiR-199a-3p was also shown to post-transcriptionally attenuate the expression of Runx1, a key regulator during the megakaryopoiesis process [77].

### 2.7. Other Functions of miR-199a

Since binding of miRNA occurs via the 3′ UTR of its targets, altered expression of 3′UTR was proposed to be an approach for development of miRNA-based gene therapy [78]. Transgenic mice over-expressing the 3′UTR of versican, a direct target of miR-199a-3p, resulted in increased fibronectin, due to enhanced binding of miR-199a-3p to versican, and thus, reduced binding to its other targets, specifically fibronectin. The study was extended to mouse breast carcinoma cells, and 3′UTR of versican again decreased the expression of miR-199a-3p, resulting in decreased inhibition on its putative target RB1. This gave rise to reduced tumor size [79].

In mouse obstructive jaundice liver, both miR-199a-3p and -5p were up-regulated. In addition, miR-199a-5p was significantly up-regulated in the intrahepatic bile duct [80]. In endometriosis affected women, miR-199a was down-regulated and affected the invasive ability of endometrial stromal cells (ESCs), partly through IKKβ/NFκB pathway suppression and reduced IL-8 expression [81]. The effects of miR-199a in ESCs also include inhibition of adhesion and invasion by directly targeting IKKβ and the inactivation of the NFκB signaling pathway [82].

MiR-199a shows various expression patterns and functions in different animal models. It was down-regulated in lipoplysaccharide-induced mouse acute lung injury [83]. MiR-199a-3p was also down-regulated in streptazotocin-inducd diabetic retinopathy in rats [84]. After spinal cord injury, rats that received cycling exercise showed decreased expression of miR-199a-3p with increased expression of both mRNA and protein of its target mTOR. The mTOR was involved in the activity-dependent plasticity in injured spinal cord [61]. After 3-nitropropionic acid preconditioning in rat brain, miR-199a showed reduced expression, indicating possible roles in the formation of cerebral ischemic tolerance through its target *Sirt1* [85]. Exposure to endocrine disrupting chemical nonylphenol, resulted in decreased expression of miR-199a-5p in mice Sertoli cells [86]. Studies in stroke-dependent brain tissue showed that MRP1 was a protective factor against stroke, and under direct regulation of miR-199a-5p [87]. In mouse kidney, expression of miR-199a-3p was up-regulated after renal ischemia perfusion injury [88].

## 3. Conclusion

**Figure 2:**
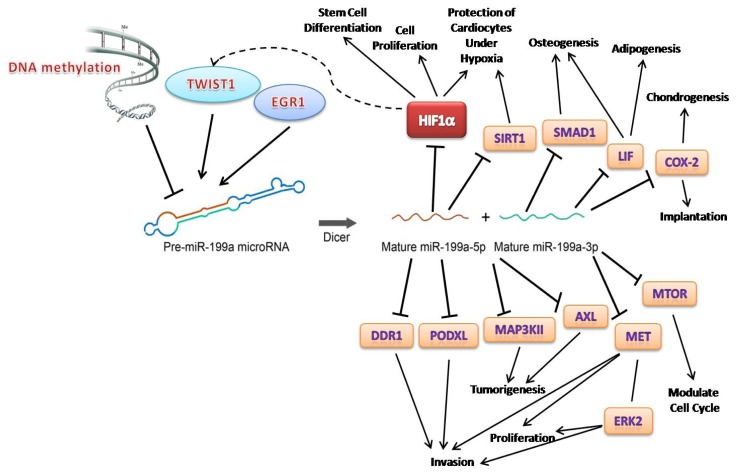
Summary of key factors relating to miR-199a and its functions on different targets. “ 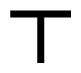
” arrows indicate inhibitory effects, while “→” arrows indicate activate effects.

It is obvious miR-199a displays extensive variability in its expression during tumorigenesis, in various diseases and from embryonic development to cell differentiation. Its two mature forms, miR-199a-3p and -5p, behave differently and have unique targets, probably due to their different seed regions. MiR-199a-3p is deregulated primarily during tumorigenesis and hepatitis, while miR-199a-5p appears be related to cardiomyocyte function and hypoxia condition (Figure 2).

Studies of one particular miRNA can be very complicated, not only because its expression pattern varies from tissue to tissue, but also because of the diverse characteristics of its targets (Figure 2). For example, HIF-1α is a direct target of both miR-199a-3p and -5p. However, the consequences of the interaction of HIF-1α with the two miRNAs are quite different. Under the effect of miR-199a-3p in liver cancer cells, HIF-1α was found to be involved in cellular proliferation and cancer growth [32]. On the other hand, miR-199a-5p protects cardiomyocyte from being damaged under low oxygen conditions [66]. The regulatory outcome is more complex since in cancer cells, HIF-1α can bind the *TWIST1* promoter directly and control its expression. Since TWIST1 controls miR-199a expression, HIF-1α and TWIST1 may form a loop with miR-199a being an intermediate molecule [89]. This hypothesis requires further validation to enhance our understanding of the regulation of miR-199a.

Since the function of a miRNA is defined and realized by its targets, it is especially important to investigate its targets globally and not to limit to one type of disease or biological process. This recognition of miRNA biology is particular important since our current knowledge of miRNAs is structure-based and not function-based. Consequently in-depth studies of different miRNAs in biology-based systems are necessary, and attempts to generalize the action of any miRNA are dangerous. This review documents the complexity of miRNA biology and suggests future directions for miRNA research.

## Figures and Tables

**Table 1:** miR-199a regulation and function in human cancer.

Cancer	miR-199a Expression	miR-199a Involved Biological Processes	Validated Targets #	References
Renal cell cancer (RCC)	Down-regulated in RCC cells and tissues		GSK-3β (protein only)	[26]
Gastric cancer *	miR-199a-3p up-regulated in patients with recurrence, miR-199a up-regulated in gastric cancer/metastatic tissues/Japanese gastric cancer tissues	Promotes proliferation and metastasis, progression-related	MAP3K11 (protein only)	[27–29]
Biliary tract cancer	miR-199a-3p up-regulated during malignancy			[30]
Human hepatocellular carcinoma (HCC)	miR-199a down-regulated	Anti-proliferation, anti-growth of HCC, PAK4/Raf/MEK/ERK pathway, anti-invasion modulator of cell cycle	HIF-1α (protein), PAK4 (protein), CD44 (mRNA), DDR1(mRNA), mTOR (mRNA)	[20,31–36]
Hepatoblastoma	miR-199a up-regulated			[37]
Osteosarcoma	miR-199a down-regulated in cells and tissues	Anti-proliferation, anti-migration and affect cell cycle	MET? mTOR? STAT3?	[38]
Esophageal adenocarcinoma	miR-199a-3p and -5p up-regulated in worse survival patients			[39]
Testicular germ cell tumors	miR-199a-3p and -5p down-regulated in cells and tissues	anti-invasion, anti-migration, anti-proliferation	PODXL (mRNA)	[40]
Breast Cancer	miR-199a down-regulated in tissues			[41]
Sezary Syndrom (T-cell lymphoma)	miR-199a-3p up-regulated	Anti-apoptosis	EVL (host gene of miR-342)	[42]
Ovarian cancer *	miR-199a-2 up-regulated in ovarian cancer stem cells; miR-199a down-regulated in serous ovarian cancer tissues	IKKβ/NFκB pathway, Poor prognosis related, tumor progression related, anti-tumor progression and chemoresistance	IKKβ (protein)	[7,43–46]
Melanoma	miR-199a up-regulated in older adults (>60)	TLR-MyD88-NFκB pathway		[47]
Uveal melanoma	miR-199a-3p and -5p up-regulated during metastasis			[48]
Bladder cancer	miR-199a-3p down-regulated in cells and tissues	Tumor suppressive	KRT7 (mRNA)	[49]
Bronchial squamous cancer	miR-199a up-regulated at a specific stage			[50]
Cervical Cancer	miR-199a up-regulated	Pro-proliferation of cells		[51]
Acute myeloid leukemia	miR-199a up-regulation in worse survival			[52]

* Suggested to be a diagnostic marker;

# “Protein only” means the target was confirmed to be affected only at translational level with no mRNA change;

“Protein” means the target was confirmed to have changes on protein expression but no test on mRNA level changes were conducted; “mRNA” means both mRNA and protein levels were proved to be changed; ? The targets were not verified to be directly inhibited by miR-199a.
